# Navigating per- and polyfluoroalkyl substances in organic waste streams: Sources, challenges and implications for circular economy recycling – A mini-review

**DOI:** 10.1177/0734242X251371421

**Published:** 2025-09-18

**Authors:** Lívia Hökl, Anke Bockreis

**Affiliations:** Unit of Environmental Engineering, Department for Infrastructure, University of Innsbruck, Innsbruck, Austria

**Keywords:** Biowaste, solid waste, recycling, closed-loop recycling, persistent pollutants

## Abstract

Waste management systems face considerable environmental and public health challenges as per- and polyfluoroalkyl substances (PFAS) emerge as major contaminants. These persistent chemicals have been detected in various waste streams, including industrial effluents, municipal solid waste, and wastewater. This article aims to identify where PFAS have been detected in biogenic waste streams, their implications for waste treatment, and highlight areas where data gaps exist and measurement methods are lacking. The literature reviewed suggests that most biological treatment methods used in waste treatment are ineffective in removing PFAS, with incineration emerging as the only reliable means of degradation. Conventional treatments do not adequately remove PFAS, so they persist in most recycled organic end products and are used in agriculture, such as compost and biosolids. PFAS re-enter the cycle and potentially contaminate agricultural land, raising questions about the sustainability and safety of recycling products and materials containing PFAS. However, data on their presence in biowaste, where food contact materials (FCMs) have been identified as a major source of PFAS, remain limited or non-existent. This knowledge gap is of particular interest given the widespread use of PFAS in food packaging and cookware. These findings highlight the need for regulatory action to address the PFAS contamination. Key recommendations include: (1) the implementation of restrictions on the use of PFAS in products with high consumer contact, especially FCMs, (2) the development of closed-loop recycling systems, and (3) the adoption of standardised analytical methods for the detection of PFAS in different waste streams and environmental matrices.

## Introduction

Per- and polyfluoroalkyl substances (PFAS) have attracted considerable attention in recent years due to their widespread presence in consumer products and the environment, their persistence, and their potential adverse effects on human health (Tavasoli et al., 2021). These synthetic compounds have carbon chains in which hydrogen atoms are either completely replaced by fluorine atoms (perfluoroalkyl substances) or partially replaced (polyfluoroalkyl substances) and are linked to different functional groups (Buck et al., 2011). The high strength of these carbon–fluorine bonds (C–F bonds) gives PFAS their stability and resistance to various forms of degradation. In addition, PFAS possess both lipophobic and hydrophobic properties (Al Amin et al., 2020). Although PFAS are known for their chemical stability and environmental persistence, these properties primarily apply to fully fluorinated, so-called terminal PFAS such as perfluorooctanoic acid (PFOA) or perfluorooctanesulphonic acid (PFOS). However, many PFAS compounds found in the environment are structurally less stable precursor compounds. These precursors can undergo transformation processes (e.g. oxidation, photolysis, hydrolysis), forming highly stable terminal PFAS as end-products. Once these terminal PFAS are present, they are no longer subject to further transformation, which highlights their role as persistent contaminants (O’Connor et al., 2022).

The discovery of PFAS dates back to the mid-20th century, when they were first synthesised for a variety of industrial applications (Clara et al., 2008). PFAS are used in a wide range of applications, including fire-fighting foam, cookware, food packaging, fabrics, and plastic products (Bolan et al., 2021).

Their extensive use has led to the widespread distribution of PFAS in the environment, raising concerns about their long-term effects on ecosystems (Lenka et al., 2021). PFAS are found in different parts of the environment, such as water, soil, and air (LfU Bayern, 2024). They enter the environment through various pathways, including industrial and wastewater treatment plant effluents, landfill leachates, and the degradation of consumer products (Stoiber et al., 2020). Once released, PFAS can persist for decades (Brunn et al., 2023), travel long distances (Evich et al., 2022; Galloway et al., 2020), and contaminate remote areas (Kourtchev et al., 2024; Pfotenhauer et al., 2022). Their persistence and mobility pose significant challenges to environmental remediation efforts (Bolan et al., 2021).

Data on PFAS in waste streams are still evolving. Studies have shown the presence of PFAS in organic waste streams (Choi et al., 2019; Tavasoli et al., 2021; Timshina et al., 2024), recycling products (Reinhart et al., 2023; Tumu et al., 2024) and their bioaccumulation in food chains (Adu et al., 2023; Focker et al., 2022; O’Connor et al., 2022; Sanchez-Hernandez et al., 2024).

This accumulation, resulting from the pre-existing presence of PFAS in organic waste streams and recycling products, can have adverse effects on the health of organisms, the ecosystems in which they live and ultimately humans (Adu et al., 2023; O’Connor et al., 2022; Sanchez-Hernandez et al., 2024).

The potential impact of PFAS on material recovery and therefore on circular economy practices is significant. Recycling of materials containing PFAS can result in the presence of PFAS in recycled products, raising concerns about the safety and viability of such practices (Reinhart et al., 2023). This contamination poses health and environmental risks (Reinhart et al., 2023), calling into question the continued relevance of material recycling in the context of PFAS. Consequently, the development of effective strategies to deal with PFAS contamination is essential to promote sustainable resource use and advance the circular economy.

In 2024, Austria implemented a comprehensive PFAS action plan, providing guidance on PFAS management, improving information exchange, and promoting a coordinated national approach (Kaiser et al., 2024). Internationally, several European countries, including Germany, Denmark, the Netherlands, Norway, and Sweden, are developing an European Union (EU)-wide restriction process for PFAS, that aims to ban more than 10,000 individual PFAS (European Chemicals Agency, 2023). This joint effort, which was submitted to the European Chemicals Agency on 13 January 2023 (European Chemicals Agency, 2023), is an important step towards harmonised regulation of PFAS in the EU. Alongside these initiatives, the EU is also establishing concrete PFAS limit values. The new Packaging and Packaging Waste Regulation (PPWR), which will come into effect in August 2026, sets strict maximum levels for PFAS in food packaging and contact materials (European Parliament and Council of the European Union, 2024). In the area of drinking water, the EU Drinking Water Directive now mandates maximum values of 0.1 µg L^−1^ for the sum of 20 PFAS and 0.5 µg L^−1^ for total PFAS (European Parliament and Council of the European Union, 2020). In the United States, the Environmental Protection Agency (EPA) introduced nationwide PFAS drinking water standards in 2024. For the two most prevalent PFAS compounds, PFOA and PFOS, the limit is set at 0.004 µg L^−1^ each. Additionally, PFAS, such as perfluorohexanesulphonic acid (PFHxS), perfluorononanoic acid (PFNA) and hexafluoropropylene oxide dimer acid (HFPO-DA), were assigned limits of 0.010 µg L^−1^ (U.S. EPA, 2024).

Through a detailed analysis, the article aims to contribute to a better understanding of PFAS contamination in biogenic waste and to inform strategies for effective waste management and circular economy practices.

In this review, ‘biowaste’ is defined as the organic fraction of municipal solid waste that is separately collected, including kitchen and food waste as well as garden waste. Other biogenic waste types, such as agricultural residues or industrial organic waste are not included in the primary scope, but may be referenced for comparison or to highlight PFAS-related issues in the broader context of waste management.

## Methodology

To ensure a comprehensive review of the existing literature, a systematic search was conducted in several academic databases, including Google Scholar, SCOPUS, and EBSCO. The search strategy involved the use of specific keywords in combination with Boolean operators to maximise the retrieval of relevant studies. The keywords were selected based on their direct relevance to the research topic and were structured to ensure broad coverage of the field. The search terms included: per- and polyfluoroalkyl substances OR PFAS AND biowaste OR organic waste OR food waste OR treatment plants OR anaerobic digestion OR composting OR recycling OR food cycle.

To further enrich the review, and ensure that no relevant studies were missed, a snowball sampling technique was used. This involved examining the reference lists of the initially selected articles to identify additional relevant publications. Each newly identified publication was then subjected to the same inclusion criteria to maintain consistency throughout the review process. To ensure the quality and relevance of the selected studies, the following inclusion criteria were applied: only articles published in peer-reviewed journals were considered; studies had to have been published within the last 20 years; and only articles written in English or German were included. The search was carried out between March and September 2024.

## Limitations in PFAS analysis and comparability

The comparability of PFAS levels and studies is complex due to the diverse nature of these compounds and the evolving analytical methods used to detect them. According to the OECD (2018), approximately 4730 PFAS compounds have been identified, but the actual number is likely to be higher, with estimates suggesting that the total PFAS universe exceeds 10,000 compounds (Glüge et al., 2020; OECD, 2021).

Various techniques are used to analyse PFAS, including liquid chromatography-mass spectrometry, targeted and non-targeted high-resolution mass spectrometry and gas chromatography-mass spectrometry (Al Amin et al., 2020), accompanied by a variety of extraction methods, each of which has its strengths and limitations. This problem is amplified by the fact that studies often quantify different sets of PFAS compounds, with only 20–30 compounds routinely monitored in regulatory programmes (European Parliament and Council of the European Union, 2000; U.S. EPA, 2021b) and validated methods available for just 100–200 PFAS (Trier et al., 2025).

In addition to the targeted analysis of individual PFAS, summarised methods are gaining importance. Techniques such as the determination of adsorbable organic fluorine or extractable organic fluorine allow the estimation of the total burden of organofluorine compounds, independent of the identity of individual substances. The total oxidizable precursor (TOP) assay is another approach that converts PFAS precursors, compounds with non-fluorinated parts that can transform into more persistent perfluoroalkyl acids (PFAAs), into terminal PFAS. These terminal PFAS are highly persistent and do not degrade further under environmental conditions. By oxidising the precursors to form a terminal compound, the TOP assay provides a more comprehensive picture of PFAS contamination (Baqar et al., 2025). However, these methods cannot identify specific PFAS and are subject to their own uncertainties.

As noted above, alongside the variety of analytical techniques, a number of sample pre-treatment methods are used in PFAS analysis, each of which can have a significant effect on both the representativeness of the sample and the reliability of the analytical results. The choice of pre-treatment has a direct impact on the selectivity and sensitivity of PFAS detection. Commonly used pre-treatment approaches include solid-phase extraction, solid–liquid extraction, liquid–liquid extraction and ultrasonic-assisted extraction (Baqar et al., 2025).

As shown in Table 1, this leaves over 9800 compounds undetectable due to the lack of analytical standards or methodological limitations. In addition, single-compound analysis does not capture the full spectrum of PFAS present in a sample because it only targets a limited number of well-characterised substances and overlook precursor compounds that can degrade to terminal PFAS (Al Amin et al., 2020). This is further complicated by inconsistencies in analytical methods, which differ in their sensitivity and scope. The limited availability of analytical standards, which exist for less than 1% of known PFAS compounds, further amplifies these issues. It is important to consider these limitations when interpreting and comparing PFAS data from different studies and to work towards standardising analytical methods for more consistent and comprehensive PFAS monitoring and assessment.

**Table 1.** PFAS detection scope and analytical limitations.

Category	Estimated PFAS	Description
Total known PFAS	~4730	Identified PFAS compounds listed by the OECD (2018)
Estimated total PFAS	>10,000	Includes structurally similar compounds and uncharacterised transformation products (Glüge et al., 2020; OECD, 2021)
Routinely monitored PFAS	20–30	PFAS included in regulatory programmes (e.g. U.S. EPA; EU Water Framework Directive)
Analytically detectable PFAS	100–200	Compounds with validated standards and methods (Trier et al., 2025)
PFAS not routinely detectable	>9800	Majority lack standards or methods (ultrashort-chain, branched isomers, precursors)

EPA: Environmental Protection Agency; PFAS: per- and polyfluoroalkyl substances.

## PFAS in waste management

PFAS, including compounds such as PFOA and PFOS and/or their substitutes (e.g. HFPO-DA, fluorotelomer alcohol (FTOH) and fluorotelomer betaine), are commonly found in consumer products and packaging materials. PFAS are used in various industrial applications, such as the manufacture of non-stick coatings, waterproof textiles, aqueous film forming foams and biocides (Kaiser, 2022). They are also found in household products such as food packaging, stain resistant fabrics and cleaning products (Bolan et al., 2021), ultimately contributing to the PFAS burden faced in waste management.

The implementation of the waste hierarchy faces major challenges due to the presence of persistent organic pollutants (POPs), in particular PFAS, in waste streams. The five-tier waste hierarchy set out in the EU Waste Framework Directive (Directive 2008/98/EC) prioritises waste prevention, followed by preparation for reuse, recycling, other recovery and finally disposal. However, this fundamental principle of waste management faces substantial obstacles when dealing with PFAS. The unique properties of POPs, including PFAS, make them difficult to manage within the traditional waste hierarchy.

At the prevention and reduction level, it is difficult to eliminate PFAS already in circulation because many have been in use for decades and are highly persistent in the environment (Bolan et al., 2021; Hamid et al., 2018). In addition, there are few alternatives that offer the same unique combination of chemical stability, water and grease resistance and thermal durability, making substitution difficult in many industrial applications. Re-use and recycling pose a risk of unintentional spread of PFAS and other persistent substances such as heavy metals and microplastics (Lahl and Zeschmar-Lahl, 2024; Staplevan and Hai, 2024; Stoiber et al., 2020; Thakali and MacRae, 2021). These substances are resistant to degradation and can be released into the environment through various recycling pathways. For example, the recycling of PFAS-containing paper can lead to cross-contamination, as concentrations of up to 971 µg kg^−1^ have been found in virgin and recycled paper and cardboard (Langberg et al., 2024). Jovanović et al. (2024) showed that primary PFAS contamination of paper intended for the production of food packaging happens during the recycling process. In wastewater influent, studies have reported a wide range of total PFAS concentrations from as low as 0.00098 µg L^−1^ to as high as 66.9 µg L^−1^ (Gallen et al., 2018; Moneta et al., 2023), with variation attributed to different water sources and uses. Traditional wastewater treatment processes, such as those used in EU and United States, are largely ineffective in removing PFAS (Tavasoli et al., 2021). The implementation of a fourth treatment stage (e.g. granular activated carbon, ion exchange or reverse osmosis) could significantly improve PFAS removal (O’Connor et al., 2022). However, these processes do not destroy PFAS, they simply transfer them from the treated water to another medium such as spent carbon, ion exchange resins or concentrated brine. The resulting PFAS-laden concentrates still require proper management to prevent them from being released back into the environment (O’Connor et al., 2022).

Due to the current insufficient removal of PFAS from the wastewater influent, PFAS are also found in wastewater effluent, where their concentrations vary widely, with studies reporting ranges between 0.02 and 107 µg L^−1^ (Gallen et al., 2018; Moneta et al., 2023) with PFOA being the most commonly detected compound in wastewater effluents, as shown by Loos et al. (2013). Notably, these concentrations often far exceed the EU Drinking Water Directive’s limit both of 0.1 g L^−1^ for the sum of 20 PFAS and 0.5 µg L^−1^ for total PFAS (European Parliament and Council of the European Union, 2020). Although these matrices are difficult to compare, a comparison nevertheless provides a useful perspective. In sewage sludge, PFAS can accumulate at different concentrations, depending on the wastewater sources and the treatment processes used (O’Connor et al., 2022). Studies have reported total PFAS concentrations in sludge ranging from 16 to 204 µg kg^−1^ (Higgins et al., 2005; Tavasoli et al., 2021; Zhang et al., 2013).

Energy recovery from the incineration of PFAS-containing wastes is the subject of intense debate. On the one hand, studies show PFAS in leachate (Vargette et al., 2023), ash and flue gas (Björklund et al., 2023, 2024) and the formation of potentially harmful products of incomplete combustion (PICs; Björklund et al., 2023, 2024; Weitz et al., 2024). On the other hand, studies report complete destruction of PFAS under conditions similar to those in municipal waste incinerators (860°C for 2 seconds) operating in the EU (Gehrmann et al., 2024).

Landfilling, the least preferred option in the waste hierarchy, is unsustainable due to the long-term risks of ecosystem contamination, food chain effects and groundwater pollution. The presence of PFAS in landfill leachate has been documented in different regions, with European countries reporting concentrations between <0.001 and 1.80 µg L^−1^ (Hamid et al., 2018) and American landfills showing elevated levels, ranging from 2 to 29 µg L^−1^ (Dasu et al., 2022). In addition, certain PFAS, especially volatile compounds such as 8:2 FTOH, can evaporate from landfills and enter the atmosphere, either through landfill gas or PFAS-laden bioaerosols (Stoiber et al., 2020). These airborne PFAS can travel considerable distances and may undergo chemical transformations before being deposited in the environment (Li et al., 2023).

In the current context, PFAS are being transported into environmental compartments, including the food chain, as a result of widespread use and inadequate treatment methods.

## Occurrence and distribution of PFAS in biowaste

PFAS in biowaste are an emerging concern due to their widespread use and environmental persistence (Bolan et al., 2021; Lenka et al., 2021). Biowaste refers to biodegradable organic material that can undergo biological degradation and is generated in domestic or commercial settings, including food and kitchen waste, yard and garden waste and agricultural waste. Biowaste can become contaminated with PFAS through several pathways, including the application of contaminated biosolids to agricultural land (Pozzebon and Seifert, 2023; Saliu and Sauvé, 2024), atmospheric deposition (Evich et al., 2022) and the use of products containing PFAS (Stoiber et al., 2020). Once present in biogenic waste, PFAS can persist and resist degradation (Lazcano et al., 2019; Stoiber et al., 2020), leading to their accumulation in compost (Brändli et al., 2006; Choi et al., 2019; Kupper et al., 2008; Munoz et al., 2022; Timshina et al., 2024) and digestate (Kupper et al., 2008), threatening the entire circular food system (Thakali and MacRae, 2021).

The distribution of PFAS within these waste streams varies depending on factors such as the source of the waste, regional waste management practices, and environmental conditions (Bolan et al., 2021). Understanding the occurrence and distribution of PFAS in biogenic waste is crucial for developing effective strategies to manage these chemicals and mitigate their impact on human health and the environment.

### Sources of PFAS contamination in biowaste

#### Food waste

PFAS have been consistently found in food, including vegetables, grain crops, livestock and fish, due to exposure in contaminated environments and migration during food processing and packaging (Death et al., 2021; Fair et al., 2019; Liu et al., 2019). Although data on PFAS levels in food are available, there is limited data on PFAS concentrations in food waste (Table 2). However, the presence of PFAS in the original food products and the ability of PFAS to leach from packaging and food contact materials (FCMs) suggests that they are likely to be present in food waste as well. Recent studies have provided some insight into the extent of PFAS contamination in food waste. In a study of food waste, Timshina et al. (2024) detected total PFAS at concentrations below 1 µg kg^−1^, confirming the presence of these chemicals. A comprehensive screening of food waste by Thakali et al. (2022) detected elevated levels of specific PFAS compounds, including perfluorobutanoic acid (PFBA) in the range of 0.87–7.60 µg kg^−1^ dry weight (dw), PFHxS at concentrations of 0.58 and 0.70 µg kg^−1^ dw and PFNA at a concentration of 3.08 µg kg^−1^ dw.

**Table 2.** Selected input source of PFAS in biowaste.

Source	No. PFAS tested	PFAS concentration (µg kg^−1^ dw)	References
Food waste	40	<1	Timshina et al. (2024)
	17	PFBA: 0.87–7.60 PFHxS: 0.70; 0.59^a^ PFNA: 3.08	Thakali et al. (2022)
FCM (leachate)	40	Mean: 1380	Timshina et al. (2024)
FCM	21	3.2–377	Wang et al. (2024)
FCM (paper tableware)	—	6:2 FTOH: 499^b^	Yuan et al. (2016)
Collection aid (paper)	—^c^	9–49	Minnesota Pollution Control Agency (2022)

TOF: total organic fluorine; FTOH: fluorotelomer alcohol; dw: dry weight; FCM: food contact material; PFAS: per- and polyfluoroalkyl substances; PFBA: perfluorobutanoic acid; PFHxS: perfluorohexanesulphonic acid; PFNA: perfluorononanoic acid.

a Measured in two samples.

b Measured in one sample.

c TOF analysis.

#### Food contact materials

The use of PFAS in FCMs has emerged as a major concern due to their potential to contaminate biowaste streams and, consequently, the environment (Wang et al., 2024). FCMs, particularly those designed to resist grease and moisture, have been found to contribute greatly to PFAS levels in food waste, often exceeding the contribution from the food itself. This is particularly evident in PFAS-treated paper and cardboard containers, which are widely used in fast food packaging, takeaway containers and other disposable food packaging items (Wang et al., 2024). Langberg et al. (2024) analysed for 37 types of PFAS in recycled paper and cardboard and found 14 PFAS compounds with concentrations ranging from 0.4 to 971 µg kg^−1^. According to this study, virgin paper products intended for food contact may have intentionally added PFAS for water and grease resistance. Choi et al. (2019) found that compost derived from food waste containing FCMs had higher concentrations of PFAS (28.7–75.9 µg kg^−1^) compared to compost derived from food or garden waste alone (2.38–7.60 µg kg^−1^). In another study, Timshina et al. (2024) reported PFAS concentrations of up to 1380 µg kg^−1^ in leachate from used compostable FCMs. In addition, the variability in PFAS content between different types of FCMs is considerable. Wang et al. (2024) observed PFAS concentrations ranging from 3.2 to 377 µg kg^−1^ in different FCMs, highlighting the inconsistent use of PFAS in different products. This variability also extends to alternative materials. Paper tableware made from sugarcane and reed pulp fibres was found to contain 6:2 FTOH at a concentration of 499 µg kg^−1^ (Yuan et al., 2016). These findings suggest that even materials marketed as environmentally friendly alternatives may contain considerable levels of PFAS.

In addition to direct contamination from FCMs, PFAS can also leach into food during storage or preparation, indirectly contaminating food waste (Begley et al., 2008; Lerch et al., 2023). This leaching process further complicates the issue of PFAS in biowaste streams. Migration of PFAS from packaging into food not only increases human exposure but also contributes to the overall PFAS burden in biowaste (Glenn et al., 2021).

It is important to note that while these studies focused primarily on paper and cardboard containers, fluoropolymers, produced using PFAS, are also commonly used in non-stick coatings on cookware and bakeware (Begley et al., 2008; Lerch et al., 2023). Since PFAS are used in the production process, these materials represent another potential source of PFAS exposure and contamination, albeit through different mechanisms than disposable FCMs (Lerch et al., 2023). Even though final products usually contain only traces, the formation of fluorocarbons during thermal treatment of fluoropolymers (Ellis et al., 2001), as well as the use and emission of PFAS as polymer processing aids (Lohmann et al., 2020), is still a cause for concern.

#### Garden waste

Information on PFAS in garden waste is limited compared to other sources such as industrial discharges, wastewater and consumer products. To our knowledge, no studies have focused specifically on PFAS concentrations in garden waste alone. Although some studies have examined compost derived from garden waste (Lazcano et al., 2020; Saha et al., 2024), data on PFAS levels in the original input material are still lacking. Nevertheless, it is reasonable to assume that PFAS are present in garden waste. Potential pathways of contamination include the use of contaminated soil amendments (Pozzebon and Seifert, 2023; Saliu and Sauvé, 2024), irrigation with PFAS-laden water (Focker et al., 2022; Sanchez-Hernandez et al., 2024) and atmospheric deposition (Evich et al., 2022). Although there is no direct one-to-one correlation between PFAS levels in soil and PFAS levels in garden waste, the evidence suggests that they are likely to be linked, as PFAS in soil can be taken up by plants and accumulated in organic matter (Adu et al., 2023; Gobelius et al., 2017), which at some point becomes garden waste.

#### Collection aids

Collection aids, such as compostable bioplastic bags or paper bags used for biowaste collection, are also a potential source of PFAS contamination in biowaste streams. Although direct studies of collection aids are limited, a study by the Minnesota Pollution Control Agency (2022) found that paper garden waste bags contained low levels of PFAS, with total organic fluorine detected in half of the samples at concentrations ranging from 9 to 49 µg kg^−1^. Although these levels are low, they still indicate a potential for PFAS to enter the system. PFAS were also found in virgin and recycled paper, which is often used to produce paper-based collection aids, at varying concentrations (0.4–971 µg kg^−1^) by Langberg et al. (2024). These substances then can accumulate through the recycling chain as noted by Brunn et al. (2023). These findings suggest that paper-based bags used for biowaste collection may contain different levels of PFAS depending on their source material. The impact of compostable bioplastic bags on PFAS levels in biowaste is another complex issue that requires further investigation. In addition, the waste management infrastructure itself poses a risk of (cross-) contamination of PFAS and potential environmental exposure. For example, high-density polyethylene bins, which are widely used for waste collection, can be contaminated during production or when recycled materials containing PFAS are used. As a result, these bins may become a source of contamination. There is also a significant risk of cross-contamination in waste collection vehicles.

The issue of PFAS contamination from collection aids, as well as the FCMs (see section ‘Food contact materials’), needs to be considered in the wider context of biowaste management, balancing the environmental benefits of recycling with the potential risks posed by contaminants.

#### Atmospheric deposition

Atmospheric transport and deposition are important pathways in the distribution of PFAS, as highlighted in recent studies by Evich et al. (2022) and Pfotenhauer et al. (2022). These persistent pollutants and their precursors are released to the atmosphere from a variety of sources, including industrial activities, commercial operations and waste management facilities (Gerardu et al., 2023). Environmental processes such as volatilisation from landfills and wastewater treatment plants also contribute to the atmospheric PFAS levels (Stoiber et al., 2020).

PFAS enter the atmosphere via complex pathways, involving both short-range transport, where PFAS are deposited close to emission sources (Dauchy, 2023), and long-range transport, facilitated in part by their extended atmospheric residence time (Evich et al., 2022; Kourtchev et al., 2024). Significant concentrations have been observed in air and precipitation samples at various geographical locations, including remote areas (Casal et al., 2017; Gewurtz et al., 2019; Kourtchev et al., 2024; Pfotenhauer et al., 2022). For example, PFAS concentrations in precipitation samples have been reported to range from 0.7 to 6.1 ng L^−1^ (Pfotenhauer et al., 2022), with some studies observing higher concentrations up to 60 ng L^−1^ for certain compounds (Pike et al., 2021). These concentrations would exceed the current drinking water limits for PFAS set by both the EU and the United States in several cases, often by a significant margin (European Parliament and Council of the European Union, 2020; U.S. EPA, 2024). The persistence and long-range transport potential of PFAS pose challenges to environmental monitoring and regulatory efforts. Deposition occurs through both wet and dry mechanisms (Pfotenhauer et al., 2022), and atmospheric transformation of PFAS precursors to more stable compounds adds complexity to the overall environmental burden (Xia et al., 2024). This transformation can occur during long-range transport (Li et al., 2023), potentially altering the chemical composition and environmental impact of PFAS. This volatility plays a crucial role in their environmental behaviour, as they can readily volatilise from contaminated surfaces and re-enter the atmosphere, creating a cyclic pattern of deposition and re-emission. This cycle greatly complicates efforts to track and mitigate PFAS contamination.

An overview of the identified sources of PFAS contamination sources in biogenic waste, including the reported concentration levels, is given in Table 2. PFAS concentrations vary significantly depending on the sample tested, with food waste showing relatively low levels (<1–7.6 µg kg^−1^ dw), whereas FCM leachate shows the highest concentrations (1380 µg kg^−1^ dw). Paper-based FCM products and collection aids fall within a wide range, highlighting the variability in contamination levels across different materials.

## Implication for biowaste treatment methods

### Composting

Composting, which converts biodegradable organic material such as garden and food waste into a nutrient-rich soil amendment, is an important waste management strategy. Although composting is a widely used method of organic waste management and it effectively stabilises organic matter and reduces pathogens (Fernández et al., 2007), its effectiveness in reducing PFAS levels remains limited (Lazcano et al., 2019). The effect of composting on PFAS concentrations varies throughout the process (Timshina et al., 2024). The often-described increase in PFAS concentrations during the curing phase could be due to several factors, including the degradation of organic matter leading to a concentration effect (Timshina et al., 2024), the transformation of PFAS precursors into more stable forms (Washington et al., 2010), or changes in the physicochemical properties of the compost affecting PFAS binding and mobility (Adu et al., 2023).

On the other hand, the composting process often involves mixing organic waste with additional materials such as garden waste and animal bedding, resulting in lower concentrations of PFAS in the final compost compared to the raw material. However, this reduction is mainly due to dilution rather than actual removal or degradation of PFAS (Thompson et al., 2023). Heat treatment and microbial activity during the composting process can potentially increase PFAA concentrations by degrading PFAS precursors (Liu and Mejia-Avendaño, 2013). Of the known precursors, FTOH, particularly 6:2 FTOH, have been identified as a significant source of perfluorocarboxylic acid (PFCA) formation during aerobic conditions such as composting. 6:2 FTOH degrades aerobically via multiple oxidation steps to form persistent PFCA. The process involves key intermediates such as 6:2 fluorotelomer aldehyde, fluorotelomer carboxylic acid and 6:2 fluorotelomer unsaturated carboxylic acid and follows two main pathways. The dominant route generates perfluorohexanoic acid and perfluoropentanoic acid, whereas secondary branches can also form PFBA and other short-chain acids (Butt et al., 2014).

Transformation of precursors has been observed to occur naturally in organic soils and biosolids (Washington et al., 2010). Although data are limited, this transformation is also expected to occur during composting. Not only does this transformation not eliminate PFAS, but it can also create more persistent and mobile forms of these chemicals. Furthermore, residual precursors in the compost can continue to degrade over time and serve as a long-term sources of contamination (Lazcano et al., 2019).

### Anaerobic (co-)digestion

Anaerobic digestion (AD) is widely used to treat and stabilise sewage sludge, crop residues, animal manure, food waste, and biowaste, while producing renewable energy in the form of biogas and recovering valuable nutrients, which are concentrated in the digestate. The use of an external carbon source makes AD an attractive biological remediation method, as it can degrade and convert a variety of organic wastes into energy-rich methane (CH_4_) while reducing pollutant loads (Choi et al., 2018). To enhance its efficiency and expand the spectrum of treatable waste, AD is often implemented in the form of co-digestion. This process involves the simultaneous digestion of several organic waste streams in a single digester, typically combining agricultural waste such as animal manure with other organic materials such as food waste or crop residues (Li et al., 2014; Zhang et al., 2013).

In the case of conventional thermophilic AD, Deligiannis et al. (2024) showed that there was no removal of PFAS, whereas Lakshminarasimman et al. (2021) found a reduction in total PFAS fluorine load in three out of five AD systems studied. In addition, the presence of PFAS in organic waste streams can potentially affect the AD process and the quality of its end products (Jiao et al., 2022; U.S. EPA, 2021a). PFAS can interfere with AD by affecting microbial communities, with varying effects depending on the specific compound and concentration (Choi and Kan, 2024; Silva et al., 2022). Some PFAS, such as PFOA and PFOS, have been found to inhibit key microbial processes such as methanogenesis and acidogenesis, thereby reducing the efficiency of AD (Jiao et al., 2022). Moreover, PFAS can be adsorbed to waste water sludge during AD, but can desorb under certain conditions (Li et al., 2021), resulting in fluctuating concentrations and making AD systems both a sink and a potential source of PFAS contamination.

### Incineration

Incineration as a waste management option involves the thermal decomposition of waste at elevated temperatures, resulting in a significant reduction in waste volume and the generation of ash, flue gases and energy (Liu et al., 2023; Vargette et al., 2023). The European Industrial Emissions Directive requires temperatures of at least 850°C for at least 2 seconds for general waste incineration (European Parliament and Council of the European Union, 2010).

The effectiveness of thermal treatment in destroying PFAS is still heavily debated. On the one hand, studies show that controlled incineration can effectively degrade certain PFAS, such as PFOA and PFOS (Stoiber et al., 2020) with destruction efficiencies of 99.99% for chlorofluorocarbons and fluoropolymers in both waste incinerators and cement kilns (Améduri and Hori, 2023; Gehrmann et al., 2024; Vargette et al., 2023) without generating low molecular weight PFAS (Aleksandrov et al., 2019). Factors influencing the fate of PFAS include temperature, oxygen levels, residence time, and furnace configuration (Hakeem et al., 2024). On the other hand, (Björklund et al., 2023) suggested that PFAS can only be effectively destroyed at temperatures above 1000°C with sufficiently long residence times.

In general, high temperatures are required to destroy PFAS due to their stability. The temperature required for PFAS destruction depends, among other factors, on the number of fluorine atoms on a given carbon and the length of the carbon chain (Reinhart et al., 2023). Low temperature regions within the incinerator can lead to the formation of PICs and possibly leads to the generation of short-chain PFAAs and/or other more stable by-products (Reinhart et al., 2023). For example, various short- and long-chain PFAS have been detected in incinerator leachates at concentrations ranging from 7.2 to 16.5 µg L^−1^ (Vargette et al., 2023). Liu et al. (2021) found a broad distribution of PFAS dominated by short-chain PFCAs and perfluorosulphonic acids when measuring PFAS in by-products from municipal solid waste incinerators. It is the incomplete destruction of PFAS that can lead to the formation of often undefined shorter-chain PFAS, which are more difficult to characterise (Winchell et al., 2021). Although the measured concentrations of PICs are much lower than those from other treatment options, the presence of PFAS in these residues still raises concerns about secondary environmental contamination through leachate and air emissions (Meegoda et al., 2022).

## PFAS contamination in recycled organic outputs

### Compost

Compost has been widely identified as a potential source of PFAS contamination (Lazcano et al., 2020). Concentrations of PFAS have been found in source-separated green and kitchen waste compost ranging from 3.4 to 35.2 µg kg^−1^ (Brändli et al., 2006). Similarly, Kupper et al. (2008) found concentrations ranging from 2.7 to 35.3 µg kg^−1^ in compost derived from similar sources, highlighting the impact of biowaste on soil and groundwater contamination. In contrast, significantly higher PFAS concentrations ranging from 26.1 to 102 µg kg^−1^ (PFOA) were reported by Sungur et al. (2020). Furthermore, Timshina et al. (2024) provided evidence of a correlation between compost maturity and PFAS concentrations. In their study, increasing concentrations were observed along the windrow ranging from 1.85 to 23.1 µg kg^−1^ and in the mature pile, with a proportional increase with curing age, ranging from 12.6 to 84.3 µg kg^−1^.

A complex interplay of factors influences the variability of PFAS levels in compost (Table 3). The composition of the feedstock plays a crucial role, with garden waste yielding lower PFAS levels than mixtures containing food waste, whereas compost derived from biosolids tends to have the highest concentrations (Lazcano et al., 2020). In addition, the composting process and time itself influence PFAS distribution, as they can lead to the transformation of PFAS compounds (Munoz et al., 2022; Saha et al., 2024).

**Table 3.** Concentrations of PFAS in the outputs of organic recycling.

Source	Origin	No. PFAS tested	PFAS concentration (µg kg^−1^ dw)	References
Compost	Green and kitchen waste	21	3.4–35.2 (mean: 6.3)	Brändli et al. (2006)
	Green and kitchen waste	4	6:2FTS/FT(UCA): 0.4–1.5^a^ PFS: 1.0–23.6 PFCA: 1.3–9.9 FOSA/FOSE: 0–0.3^b^	Kupper et al. (2008)
	—	2	PFOA: 26.1–102 PFOS: 0.21–0.65	Sungur et al. (2020)
	Food waste	40	1.85–23.1 12.6–84.3	Timshina et al. (2024)
Digestate	Green and kitchen waste	4	6:2FTS/FT(UCA): n.d.–0.9^c^ PFS: 2.0–8.6 PFCA: 2.4–6.6 FOSA/FOSE: 0.2–0.4^d^	Kupper et al. (2008)
Biosolids	Urban, rural, industrial waste water	44	4.2–910 (mean: 260)	Moodie et al. (2021)
	Small rural to big industrial waste water	24	1–3200 (mean: 108 ± 277)	Link et al. (2024)

n.d.: not detected; 6:2 FTS/FT(UCA) 6:2 fluorotelomersulphonic acid/6:2 fluorotelomer unsaturated carboxylic acid; dw: dry weight; FOSA/FOSE: perfluorooctane sulphonamide/perfluorooctane sulphonamide ethanol; PFAS: per- and polyfluoroalkyl substances; PFCA: perfluorocarboxylic acid; PFOA: perfluorooctanoic acid; PFOS: perfluorooctanesulphonic acid; PFS: sum of perfluorinated sulphonates.

a In seven samples.

b In seven samples.

c In two samples.

d In four samples.

Over the past decades, compost-containing PFAS has been applied to agricultural fields, resulting in the spread of PFAS through the environment. In Germany, Röhler et al. (2021) linked two contaminated agricultural sites to the use of compost that was mixed with PFAS-contaminated paper sludge. At one site, a maximum PFOA + PFOS concentration of 6300 µg kg^−1^ was measured (Röhler et al., 2021). The study also suggests that it could take decades for PFAS to be completely leached out of the topsoil.

### Digestate

Digestate, as the nutrient-rich end product of AD, has emerged as a promising soil amendment and fertiliser (Lin et al., 2018). It is typically present as a slurry consisting of both solid and liquid fractions, which can be further separated into fibrous solid and a nutrient-rich liquid, depending on treatment needs and plant operations. However, the presence of PFAS in digestate has raised concerns about its environmental impact and management (Thakali et al., 2022). Studies on the concentration of PFAS in digestate are limited. In one of the few studies on digestate, Kupper et al. (2008) found PFAS concentrations ranging from 4.6 to 16.5 µg kg^−1^.

PFAS levels in digestate are influenced by a number of factors. Similar to compost, the type of feedstock used in the AD process greatly influences the physicochemical properties of the digestate (O’Connor et al., 2022) and may also influence the levels of PFAS in the output, with digestate derived from biosolids or sewage sludge potentially containing higher concentrations of PFAS compared to digestate from other sources (U.S. EPA, 2021a). Digestate produced from food waste may have elevated levels of PFAS, whereas digestate produced from green waste alone may have lower concentrations, but it is not yet clear whether digestate produced from food waste contains higher, comparable or lower levels of PFAS compared to digestate produced from other feedstock sources (U.S. EPA, 2021a).

### Waste water sludge

PFAS compounds are resilient during the treatment and processing of sewage sludge into biosolids. As a result, these persistent chemicals often remain present in the final biosolid-based products that are subsequently used as fertilisers or soil amendments (Lazcano et al., 2019).

Studies by Link et al. (2024) and Moodie et al. (2021) found concentrations of 4.2–910 µg kg^−1^ and 1–3200 µg kg^−1^, respectively. Despite the voluntary phase-out of PFOS, it was generally found to be present at higher concentrations than other PFAAs in biosolid-based products (Lazcano et al., 2020).

PFAS-laden biosolids can contaminate soil, dairy, groundwater and surface water when applied to agricultural land (DeSilva et al., 2021; Pozzebon and Seifert, 2023; Saliu and Sauvé, 2024). Due to their high mobility, especially in water, PFAS have a significant potential to spread through the environment (Evich et al., 2022). They can be taken up by crops grown on treated soils (Choi et al., 2019; Liu et al., 2019) or ingested by livestock grazing on affected land (Death et al., 2021; Link et al., 2024), leading to their accumulation in the food chain. This situation raises serious concerns about food safety and the risk of human exposure (Liu et al., 2019).

Table 3 gives an overview of the PFAS concentrations in the outputs of organic recycling. The detected levels vary depending on the material and source. Compost and digestate derived from green and kitchen waste generally show lower concentrations (e.g. mean: 6.3 µg kg^−1^ dw in compost, <0.9–8.6 µg kg^−1^ dw in digestate), whereas biosolids, especially from industrial wastewater, show the highest PFAS burdens, reaching up to 3200 µg kg^−1^ dw. These variations highlight the influence of feedstock and treatment processes on PFAS contamination in recycled products. A comparison with the regulatory framework shows that even the lowest concentrations of PFAS detected are several orders of magnitude higher than the limit set by the EU drinking Water Directive (0.1 µg L^−1^ for the sum of 20 PFAS; European Parliament and Council of the European Union, 2020).

## Considerations for PFAS and recycling regulation

The management of PFAS in recycling processes is challenging due to both the complexity of these compounds and our evolving understanding. The lack of standardised measurement methods and different protocols for biowaste and other media makes it difficult to set consistent and enforceable regulatory limits. This affects not only the accuracy of PFAS quantification but also the comparability of data between studies. The issue is further complicated by the introduction of the new generation of PFAS variants, which are being used without a full understanding of their behaviour, effects and recyclability. The challenge is further compounded by the constant flow of new information on PFAS. As research progresses, new PFAS compounds are identified, and the understanding of their behaviour and impact evolves.

As PFAS move through the waste management chain, they partition into different media and transform into different degradation products (see section ‘PFAS contamination in recycled organic outputs’), making it difficult to monitor and quantify the total flow. Monitoring a wide range of PFAS, including mobile compounds and their degradation products, is essential for a comprehensive assessment of PFAS levels, including in the disposal pathways. Even though analytical methods are evolving to cover broader spectrums and detect lower and lower concentrations, they are still unable to capture the full diversity of PFAS.

It is important to consider the issue of PFAS contamination in the wider context of biowaste management and recycling. The EPA emphasises the importance of balancing the benefits of biowaste recycling with the risks posed by contaminants such as PFAS. Recycling biowaste has major environmental benefits. These include reducing greenhouse gas emissions, conserving landfill space and producing valuable compost for agricultural use. However, the widespread presence of PFAS, including in the biogenic waste stream, poses a potential risk to human health and the ecosystems (see section ‘Sources of PFAS contamination in biowaste’). This complex issue requires a holistic approach to biowaste management, taking into account both the benefits of recycling and the challenges posed by emerging contaminants.

Material recycling, although beneficial in reducing landfill waste, conserving resources and recovering nutrients, poses a risk of spreading PFAS contamination into secondary raw materials, threatening the circular economy. This can lead to bioaccumulation in the environment and potential exposure through various routes. Therefore, they can be considered as mostly non-recyclable and not in line with the circular economy. In contrast, closed-loop recycling offers better containment of PFAS, minimising the spread of contaminants into other material cycles. However, the implementation of closed-loop systems for biowaste poses challenges, including the heterogeneity and variable composition of biowaste, higher resource intensity and limited applicability for certain waste streams.

As research continues to reveal the extent and impact of PFAS contamination in biowaste streams, policymakers and waste management professionals are working together to develop strategies that maximise the benefits of biowaste recycling while minimising the risks associated with PFAS and other contaminants. The high contribution of PFAS from FCMs (see section ‘Food contact materials’) means that efforts to reduce PFAS in biowaste should focus on tackling PFAS in packaging and other FCMs. This targeted approach could lead to significant reductions in the overall PFAS levels in biowaste streams and end products.

## Conclusion and recommendations

The ubiquitous nature of PFAS contamination in different waste streams underlines the complexity of this environmental issue. However, the lack of data on the presence of PFAS in biowaste streams represents a critical information gap that hinders our understanding of the full extent of this problem. The limited data available on PFAS in biowaste suggest concentrations generally below 10 μg kg^−1^, with higher levels observed where FCMs are present. This discrepancy highlights the potential importance of FCMs as a major contributor to PFAS contamination in biowaste.

Of particular concern is the inefficacy of conventional biological treatment methods to remove PFAS from waste streams. Although thermal destruction, especially high-temperature incineration, has emerged as the most reliable degradation technique, it is not without drawbacks, including the potential formation of harmful by-products. Moreover, although the incineration of biowaste is often regarded as carbon-neutral due to the biogenic origin of the emitted CO_2_ (Beck et al., 2017), it leads to the irreversible loss of organic carbon and humic substances, which are key components for soil health and long-term ecological stability (Meissl and Smidt, 2007). This limitation underlines the urgent need for innovative treatment technologies that can effectively and efficiently degrade PFAS without creating secondary environmental risks.

A major challenge in managing PFAS contamination lies in the analytical methods currently available. The lack of standardised, comprehensive analytical techniques capable of detecting and quantifying the full range of PFAS compounds, including emerging variants and degradation products, hinders our ability to fully characterise the extent of contamination in waste streams and the environment. This analytical gap is particularly pronounced for complex matrices such as biowaste, where interfering compounds can make accurate PFAS detection and quantification difficult. In addition, the persistence of PFAS in recycled organic end products, such as compost and biosolids, raises critical questions about the long-term sustainability of current recycling practices for PFAS-containing materials. The potential for these substances to re-enter the environmental cycle through land application of contaminated materials poses a clear risk to agricultural sustainability and food safety. This cyclical contamination pathway requires a re-evaluation of material recycling strategies, particularly for products known to contain or come into contact with PFAS. To date, any disposal pathway carries a risk of PFAS, their precursors and/or their degradation products being released into other waste streams or the environment, unless efficient treatments are installed and the waste management system is adapted.

Effective strategies for the reduction of PFAS contamination should be focused on the following key measures:

1. Limit the use of PFAS: Implement regulations to significantly reduce the production and release of PFAS. Restrictions should include limiting the development of new PFAS compounds and restricting the use of PFAS to essential products where viable alternatives do not yet exist. In addition, enforce a complete ban on PFAS in products with high consumer contact and implement stricter regulations on PFAS-containing recyclates.
2. Investigate closed-loop recycling systems: Promote the development and implementation of closed-loop recycling technologies for products containing PFAS to minimise their dispersion and encourage manufacturers to design products that facilitate recycling.
3. Validate standardised analytical methods: Adopt standardised methods for PFAS analysis to increase the comparability and validity of contamination assessments across different studies and regions. This should include the development of methods to analyse PFAS in complex matrices.
